# Increased endothelial sclerostin caused by elevated *DSCAM* mediates multiple trisomy 21 phenotypes

**DOI:** 10.1172/JCI167811

**Published:** 2024-06-03

**Authors:** David M. McKean, Qi Zhang, Priyanka Narayan, Sarah U. Morton, Viktoria Strohmenger, Vi T. Tang, Sophie McAllister, Ananya Sharma, Daniel Quiat, Daniel Reichart, Daniel M. DeLaughter, Hiroko Wakimoto, Joshua M. Gorham, Kemar Brown, Barbara McDonough, Jon A. Willcox, Min Young Jang, Steven R. DePalma, Tarsha Ward, Pediatric Cardiac Genomics Consortium Investigators, Richard Kim, John D. Cleveland, J.G. Seidman, Christine E. Seidman

**Affiliations:** 1 Department of Genetics, Harvard Medical School, Boston, Massachusetts, USA.; 2 Cardiovascular Division, Brigham and Women’s Hospital, Boston, Massachusetts, USA.; 3 Weill Cornell Medicine, New York, New York, USA.; 4 Division of Newborn Medicine, Boston Children’s Hospital, Boston, Massachusetts, USA.; 5 Department of Pediatrics, Harvard Medical School, Boston, Massachusetts, USA.; 6 Walter Brendle Centre of Experimental Medicine, University Hospital, Ludwig Maximilian University of Munich, Munich, Germany.; 7 Department of Cardiology, Boston Children’s Hospital, Boston, Massachusetts, USA.; 8 Howard Hughes Medical Institute, Harvard University, Boston, Massachusetts, USA.; 9 The Pediatric Cardiac Genomics Consortium Investigators are detailed in Supplemental Acknowledgments.; 10 Section of Cardiothoracic Surgery, University of Southern California Keck School of Medicine, Los Angeles, California, USA.

**Keywords:** Cardiology, Genetics, Bone development, Cardiovascular disease, Genetic diseases

## Abstract

Trisomy 21 (T21), a recurrent aneuploidy occurring in 1:800 births, predisposes to congenital heart disease (CHD) and multiple extracardiac phenotypes. Despite a definitive genetic etiology, the mechanisms by which T21 perturbs development and homeostasis remain poorly understood. We compared the transcriptome of CHD tissues from 49 patients with T21 and 226 with euploid CHD (eCHD). We resolved cell lineages that misexpressed T21 transcripts by cardiac single-nucleus RNA sequencing and RNA in situ hybridization. Compared with eCHD samples, T21 samples had increased chr21 gene expression; 11-fold-greater levels (*P* = 1.2 × 10^–8^) of *SOST* (chr17), encoding the Wnt inhibitor sclerostin; and 1.4-fold-higher levels (*P* = 8.7 × 10^–8^) of the *SOST* transcriptional activator *ZNF467* (chr7). Euploid and T21 cardiac endothelial cells coexpressed *SOST* and *ZNF467*; however, T21 endothelial cells expressed 6.9-fold more *SOST* than euploid endothelial cells (*P* = 2.7 × 10^–27^). Wnt pathway genes were downregulated in T21 endothelial cells. Expression of *DSCAM*, residing within the chr21 CHD critical region, correlated with *SOST* (*P* = 1.9 × 10^–5^) and *ZNF467* (*P* = 2.9 × 10^–4^). Deletion of *DSCAM* from T21 endothelial cells derived from human induced pluripotent stem cells diminished sclerostin secretion. As Wnt signaling is critical for atrioventricular canal formation, bone health, and pulmonary vascular homeostasis, we concluded that T21-mediated increased sclerostin levels would inappropriately inhibit Wnt activities and promote Down syndrome phenotypes. These findings imply therapeutic potential for anti-sclerostin antibodies in T21.

## Abstract 2

Trisomy 21 chronically increases expression of *SOST*, a chromosome 17 gene encoding the Wnt-inhibitor sclerostin, that may contribute to cardiac, skeletal, and, pulmonary abnormalities.

## Introduction

Trisomy 21 (T21), the most common genetic cause of syndromic congenital heart disease (CHD), is diagnosed in approximately 5,000 newborns in the US annually. Almost half of affected infants have CHD, including 40% who have atrial, ventricular, or more severe atrioventricular septal defects (AVSDs) (1–3). AVSDs emerge from aberrant development of the atrioventricular canal, a complex process predicated on sonic hedgehog (Shh) and Wnt signaling (4). AVSDs are rare forms of CHD in the general population but occur 2,000-fold more frequently in newborns with T21 (5).

Approximately one-third of all individuals with CHD have additional malformations with variable organ involvement (6), while syndromic CHD exhibits prototypic extracardiac abnormalities. Typical T21 extracardiac manifestations include delays in developmental milestones with variable severity of neurocognitive deficits; abnormalities of craniofacial, axial, and long bones; short stature; and increased risks for osteoporosis due to progressively diminished bone mineral density. Affected children may develop endocrine and hematologic abnormalities, and those with CHD have increased risks for pulmonary hypertension. The pathways by which T21 causes these recurrent, systemic manifestations remain unknown.

Studies to illuminate the mechanisms accounting for T21 phenotypes have focused on the consequences of increased dosage for the 175 genes encoded on chromosome 21 (chr21). Comparative analyses of partial T21 in patients with and without CHD, combined with experimental models, have defined a CHD critical region encompassing 0.96 Mb with 3 protein-coding (*DSCAM*, *PLAC4*, and *BACE2*) and 4 noncoding genes (7, 8). Microarray analyses (9, 10) have identified dysregulated expression of genes encoded on chr21 and elsewhere that may also contribute to neurocognitive and immunodeficiency in T21. However, the causal molecular linkages between increased dosage of chr21 genes, genome-wide transcriptomics, and T21 cardiac and extracardiac abnormalities remain largely unknown.

We hypothesized that unbiased transcriptional analyses of CHD tissues would be informative about genes that contribute to T21 phenotypes. We compared genome-wide RNA expression in heart tissues discarded during surgical repair of T21 and euploid CHD (eCHD) with RNA expression in unused euploid donor (“healthy”) heart tissue. Expression of chr21 genes was consistently increased in T21 tissues. Among a few genes encoded elsewhere that were dysregulated in T21 CHD cardiac tissues, we identified markedly increased expression of *SOST*, located on chr17, which encodes the secreted Wnt inhibitor sclerostin. The combination of whole-tissue analyses with single-nucleus RNA sequencing revealed that T21 cardiac endothelial cell lineages were highly enriched for *SOST* expression and had downregulated Wnt target genes. Using correlation analyses to examine the expression of each chr21 gene and *SOST*, we identified a potential signaling pathway by which T21 increases sclerostin. We demonstrated that *DSCAM*, one of the 3 protein-coding genes within the CHD critical region on chr21, was necessary for the upregulation of *SOST* in T21 endothelial cells derived from human induced pluripotent stem cells (iPSCs). We infer from these observations that lifelong sclerostin-mediated impairment of Wnt signaling across multiple tissues may contribute to T21 CHD and extracardiac phenotypes.

## Results

### CHD cardiac tissue samples.

Under the auspices of the Pediatric Cardiac Genomics Consortium (PCGC), we enrolled individuals with CHD and obtained discarded cardiovascular tissue at the time of surgical repair (11, 12). From 284 enrolled probands (mean 2.8 years; range, newborn to 19.8 years), 298 CHD tissues were obtained (Supplemental Tables 1–3; supplemental material available online with this article; https://doi.org/10.1172/JCI167811DS1), including 51 tissues from 49 individuals with T21 and 236 tissues from 226 individuals with eCHD without clinically identified cytogenic abnormalities. T21 individuals were equally likely to be male (47% vs. 58%, *P* = 0.16) but were younger (mean 1.4 vs. 3.1 years, *P* = 0.002) than eCHD individuals. Atrioventricular canal CHD was more common in T21 participants (27 of 49 vs. 10 of 226, OR 25.9, *P* = 3.9 × 10^–16^), while tetralogy of Fallot was evenly distributed (2 of 49 vs. 31 of 226, OR 0.3, *P* = 0.09).

### RNA levels of genes encoded on chr21 and euploid chromosomes of T21 genomes.

**Figure 1:**
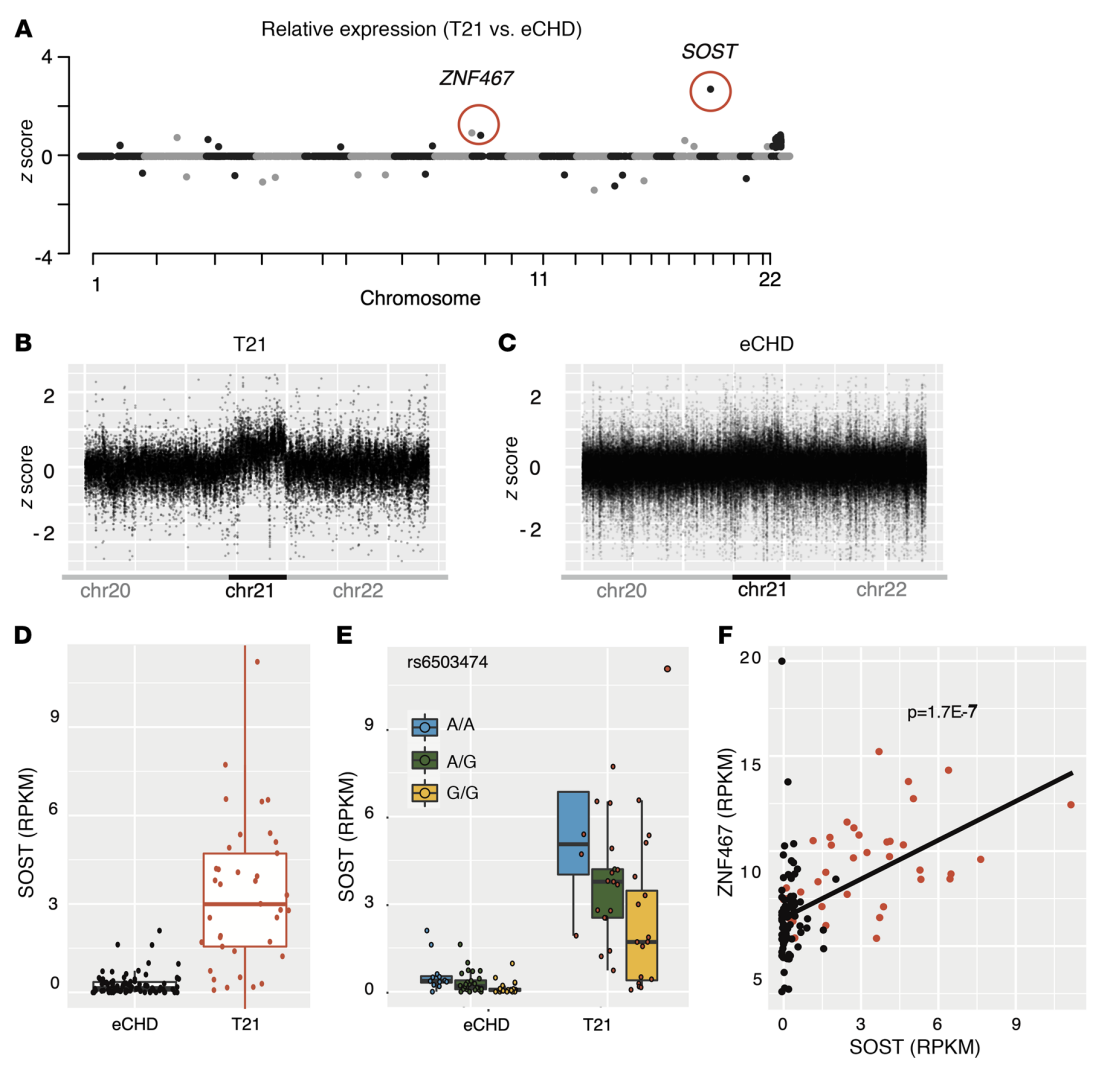
Genome-wide transcript analyses identifies increased expression of *SOST* and *ZNF467* in T21 cardiac tissues. (**A**) Graphical representation of the log_2_ fold change in RNA expression *z* score for all autosomal genes in 51 tissues from 49 individuals with T21 compared with and 236 tissues from 226 individuals with eCHD. *ZNF467* is represented by the black dot within the red circle. (**B** and **C**) Standard (*z*) scores for differentially expressed chr21 genes in T21 (**B**) and eCHD (**C**) tissues, excluding genes with 1.35 < *z* score < 0.65 or 2-tailed *t* test *P* > 0.002. (**D**) Quantification of *SOST* expression (RPKM) demonstrates 11.03-fold-higher expression in T21 than eCHD (2-tailed *t* test *P* = 1.2 × 10^–8^) RA tissues. (**E**) Quantification of *SOST* expression (RPKM) is based on rs6503474 genotypes of RA tissues from eCHD (A/A *n* = 12; A/G *n* = 29; G/G *n* = 21) and T21 (A/A *n* = 7; A/G *n* = 20; G/G *n* = 10) patients and indicates a similar effect size in T21 (42%) and euploid (35%) tissues. (**F**) Expression of *ZNF467* and *SOST* transcripts (RPKM) is correlated in eCHD (black) and T21 (red) tissues. In **D** and **E**, the box represents the first quartile (bottom) and third quartile (top) values, while the line represents 1.5 times the interquartile range beyond those values.

RNA sequencing of tissues was performed and annotated as described previously (12). CHD tissues expressed on average approximately 13,300 genes, irrespective of tissue site or genotype (Supplemental Table 4). Principal component analyses of RNA sequencing data showed clustering by tissue type, irrespective of genotype (Supplemental Figure 1). Accordingly, we deduced that T21 did not globally disrupt transcription in CHD tissues (Figure 1A).

We calculated tissue type–specific *z* scores by comparing transcript levels in each T21 tissue with levels in all eCHD tissues. Analyses of *z* scores in right atrial (RA) samples from 37 T21 and 83 eCHD individuals yielded *P* values similar to those obtained by direct comparison of transcript levels (*r* = 1.0; Supplemental Figure 2). Both methods identified 29 chr21 genes with significantly increased expression (Supplemental Table 5 and Supplemental Figure 3) and 5 non-chr21 genes with significant differential expression (Supplemental Table 6) in RA samples. We report findings combining observations made in multiple cardiac tissues.

chr21-encoded genes were expressed, on average, at 1.45-fold-higher levels in T21 tissues than in euploid tissues (Figure 1, B and C). Thirty-one of the 113 chr21 genes expressed in all cardiac tissue types were upregulated in T21 samples (fold change ≥ 1.5; *P* ≤ 4.4 × 10^–4^; Supplemental Table 7). Twenty-six genes encoded on other autosomes were differentially expressed in T21 tissues (Figure 1A, Table 1, and Supplemental Table 8). Notably, *SOST* (chr17) expression was 11-fold greater in T21 than eCHD samples (all tissue sites; Supplemental Figure 4), while other upregulated genes increased less than 2.2-fold. In RA, *SOST* expression was 12-fold higher in T21 (3.3 ± 2.5 reads per kilobase of transcript per million reads [RPKM]) than eCHD samples (0.28 ± 0.37 RPKM; *P* = 7.3 × 10^–9^; Supplemental Table 9) and did not vary with age (*r* = 0.22; *P* = 0.18; Supplemental Table 10). Bulk *SOST* RNA expression within T21 and eCHD tissues did not significantly vary by specific cardiac phenotype, sex, or oxygen status (Supplemental Table 11). *SOST* levels were comparable in RA samples from eCHD individuals when stratified by lesion. For example, *SOST* levels in RA samples from T21 subjects with AVSD (*n* = 19) were 14.8-fold higher than in euploid subjects (*n* = 5) with the same lesion (*P* = 1 × 10^–6^). Similarly, *SOST* levels in RA samples from T21 subjects without AVSD (*n* = 18) were 10.8-fold higher than in euploid tissues (*n* = 78) from subjects with the same lesion (*P* = 0.0008). *SOST* levels exceeded 2.5 RPKM in 62% of T21 samples but in none (0%) of the euploid RA samples (Figure 1D).

### SOST expression quantitative trait locus.

Comparative analyses of gene expression and genome sequences identified the polymorphism rs6503474 (normalized genotype effect size –0.25; minor allele frequency [MAF] 0.36) (13) as an expression quantitative trait locus (eQTL) for *SOST* in adult RA tissues (14). Among PCGC participants, genotypes at rs6503474 correlated with *SOST* expression in both eCHD (*r* = 0.35, *P* = 3 × 10^–3^; *n* = 61) and T21 (*r* = 0.41, *P* = 7 × 10^–3^; *n* = 37) RA tissues (Figure 1E). rs6503474 was also correlated with *SOST* expression in aorta and atrial appendage tissues in the Genotype Tissue Expression (GTEx) database (adjusted *P* values 2.5 × 10^–14^ and 3.9 × 10^–6^, respectively) (15). The genotype effect size was larger in magnitude in T21 than in eCHD tissue (–5.3 vs. –0.65, respectively), and the mean T21 *SOST* levels (RPKM) per genotype were A/A 5.8, A/G 3.9, and G/G 2.2. Overall, this eQTL explained a similar proportion of the variance in *SOST* expression in T21 and eCHD RA samples (42% vs. 35%)

### ZNF467, a transcriptional activator of SOST.

*ZNF467* (chr7), which encodes a zinc finger transcriptional activator of *SOST* (16), was also upregulated in T21 tissues (1.4-fold, *P* = 8.7 × 10^–8^; Figure 1F, Table 1, Supplemental Figure 4, and Supplemental Tables 9 and 10). Moreover, expression of *SOST* correlated with the expression of *ZNF467* in all tissues (*r* = 0.32, *P* = 1.5 × 10^–7^; Supplemental Table 12. None of the previously described eQTLs for *ZNF467* (14) correlated with expression in eCHD and T21 tissues.

### Single-nucleus RNA sequencing demonstrates endothelial cell expression of SOST and ZNF467 in human euploid and T21 atrial tissues.

**Figure 2:**
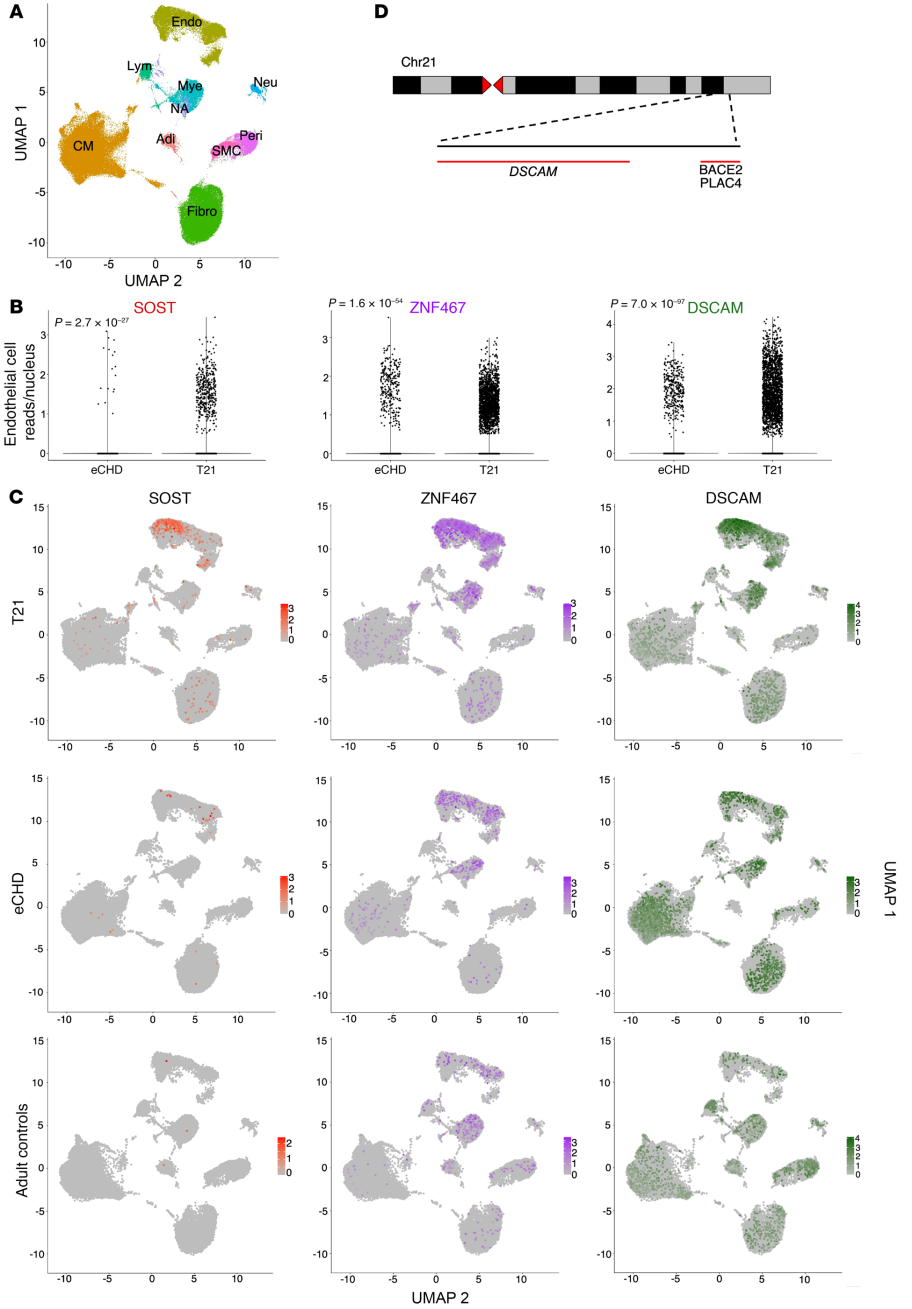
Single-nuclear RNA sequencing defines cell-type expression of *SOST*, *ZNF467*, and *DSCAM* in CHD tissues. (**A**) A uniform manifold and projection graph of single-nuclear RNA sequencing right atria from participants with T21 (*n* = 10) and eCHD (*n* = 13) and healthy adults (*n* = 12) identifies 8 major cell types (Adi, adipocytes; CM, atrial cardiomyocytes; Endo, endothelial cells; Fibro, fibroblasts; Lym, lymphocytes; Mye, myeloid cells; NA, not assigned; Neu, neural cells; Peri, pericytes). (**B**) Comparison of endothelial cell transcript levels for *SOST*, *ZNF467*, and *DSCAM* in T21 and eCHD samples. More endothelial cells demonstrate expression (≥0.01 RPKM) of *SOST*, *ZNF467*, and *DSCAM* in T21 compared with eCHD tissues: *SOST* 2.8% vs. 0.2%; *ZNF467* 12.4% vs. 2.8%; *DSCAM* 14.2% vs. 3.2%. Boxes represent the first quartile (bottom) and third quartile (top) values, while the line represents 1.5 times the interquartile range beyond those values. Each dot represents a cell with the indicated number of reads; cells with no reads are clumped at the origin. (**C**) Feature plots demonstrating variable expression of *SOST*, *ZNF467*, and *DSCAM* in different cell lineages within T21, eCHD, and healthy adult RA. Note the different scales in the heat maps; scale bars: 25 μM. (**D**) Schematic representation of the 0.96 Mb CHD critical region on chr21 with protein-coding genes *DSCAM*, *BACE2*, *PLAC4*, and *C21orf62*.

Single-nucleus RNA sequencing of RA tissues from 10 patients with T21 CHD, 13 patients with eCHD, and 12 adult participants without CHD (Supplemental Tables 13–15) defined 10 cardiac cell types (17). Cardiomyocytes, endothelial cells, and fibroblasts were the most abundant cells (Figure 2A). *SOST* expression was highest in endothelial cells, 42-fold greater than in atrial cardiomyocytes, and 6.9-fold higher in T21 compared with eCHD endothelial cells (*P* = 2.7 × 10^–27^; Figure 2, B and C). Within endothelial cells, 133 chr21 genes were expressed (mean expression ≥0.01 RPKM) in eCHD and/or T21 samples, of which 68 had T21 expression proportional to gene dosage, 49 had greater than and 16 had less than the expected 1.5-fold-increased expression (all *P* < 3.7 × 10^–4^; Supplemental Table 14). *ZNF467* was coexpressed at 2.7-fold-higher levels in T21 than eCHD endothelial cells (*P* = 1.6 × 10^–54^). Single-nucleus RNA sequencing of fetal tissues (18, 19) also reported *SOST* expression in endocardial cells, a lineage that was not captured in the CHD samples studied here.

### SOST RNA in situ hybridization confirms greater expression in T21 than in eCHD tissues.

**Figure 3:**
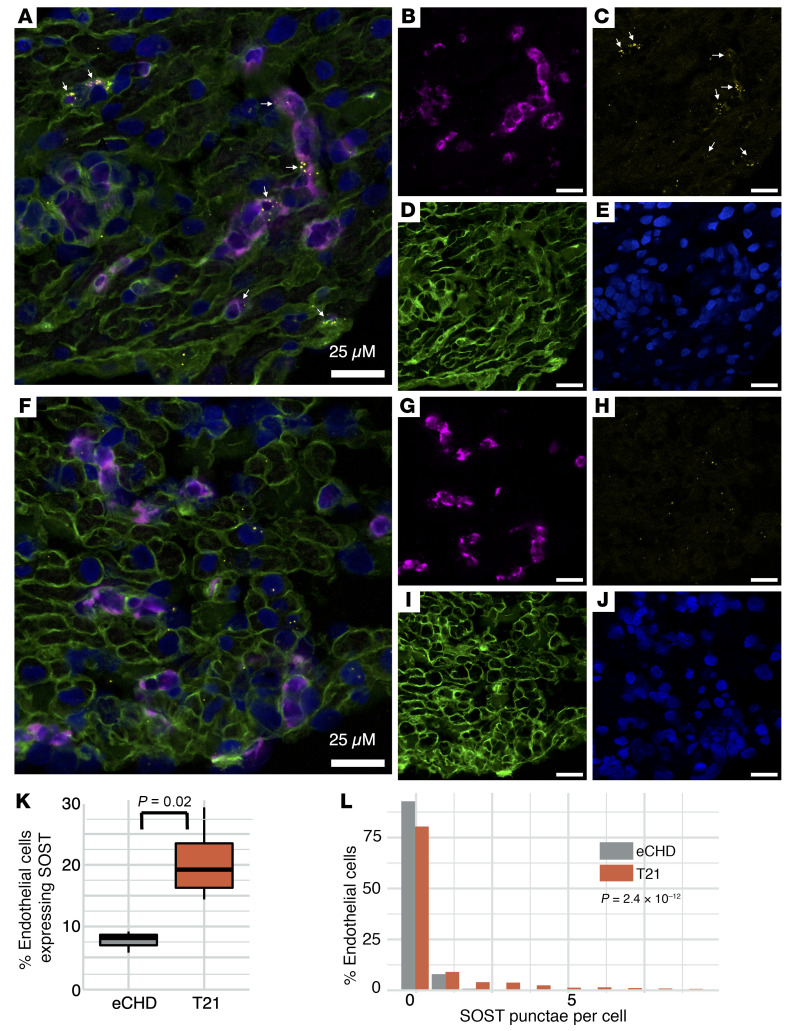
Single-molecule RNA in situ hybridization identifies prominent *SOST* expression in endothelial cells. (**A**–**J**) Fluorescently labeled RNA probes detect colocalization (white arrows) of *SOST* (red, **C** and **H**) and the endothelial cell–specific maker von Willebrand factor transcripts (*VWF*; cyan, **B** and **G**) in eCHD (*n* = 3, **A**–**E**) and T21 (*n* = 4, **F**–**J**) RA tissues. Nuclei are DAPI-stained (blue, **E** and **J**), and cell membranes are defined by wheat germ agglutinin (WGA: green, **D** and **I**). Scale bars: 25 μm; original magnification, 63×. (**K**) The proportion of endothelial cells with coexpression of *SOST* and *VWF* expression is significantly higher in T21 (20.2%) compared with eCHD (7.7%) tissues. Box represents the first (bottom) and third quartile (top) values, while the line represents 1.5-times the interquartile range beyond those values. (**L**) *SOST* expression per endothelial cell is significantly higher in T21 (*n* = 514, mean number of red puncta 0.52) compared with eCHD (*n* = 337, mean number of red puncta 0.08; 2-tailed *t* test *P* = 2.4 × 10^–12^ across all cells).

We employed single-molecule RNA in situ hybridization to confirm endothelial cell expression of *SOST*. Fluorescently labeled RNA probes for the endothelial cell–specific transcript *VWF* and *SOST* colocalized (Figure 3, A–J, Supplemental Figure 5, and Supplemental Table 16) in more endothelial cells from T21 (mean 20.2%) than in those from eCHD tissues (mean 7.7%; Figure 3K). T21 endothelial cells also had more *SOST* puncta (mean 0.52) than cells from eCHD tissues (mean 0.08, *P* = 2.4 × 10^–12^; Figure 3L and Supplemental Table 17).

### Correlation of chr21 genes with SOST in endothelial cells.

We considered the potential chr21 gene(s) responsible for elevated endothelial cell expression of *ZNF467* and *SOST* by correlative assessments of the transcript levels for these two genes and each chr21 protein-encoding gene (20) (Supplemental Table 18). We excluded chr21 noncoding RNAs, as many of these were not robustly captured by single-nucleus RNA sequencing, and assessed 133 of 224 chr21 protein-encoding genes expressed in cardiac endothelial cells. Levels of *C21orf62* — which resides approximately 5 Mb telomeric to the T21 CHD critical region, is expressed in endothelial and other cardiac cells (Supplemental Figure 6), and encodes a protein of unknown function — correlated with *SOST* but not *ZNF467* (*r* = 0.70, *P* = 2.4 × 10^–4^). Levels of *DSCAM* correlated with both *SOST* (*r* = 0.77, *P* = 1.9 × 10^–5^) and *ZNF467* (*r* = 0.69, *P* = 2.9 × 10^–4^) in T21 and eCHD endothelial cells (Figure 2, B and C). *DSCAM* resides within the CHD critical region (Figure 2D) and encodes an immunoglobulin cell adhesion molecule, and overexpression of DSCAM has been implicated in CHD (21). Consistent with their chr21 location, both *DSCAM* and *C21orf62* were expressed at higher levels in T21 than eCHD endothelial nuclei (*DSCAM*: 4.0-fold, *P* = 7.0 × 10^–97^; *C21orf62*: 1.4-fold, *P* = 1.9 × 10^–30^; Figure 2 and Supplemental Figure 6).

### DSCAM is necessary for upregulation of SOST expression in endothelial cells.

**Figure 4:**
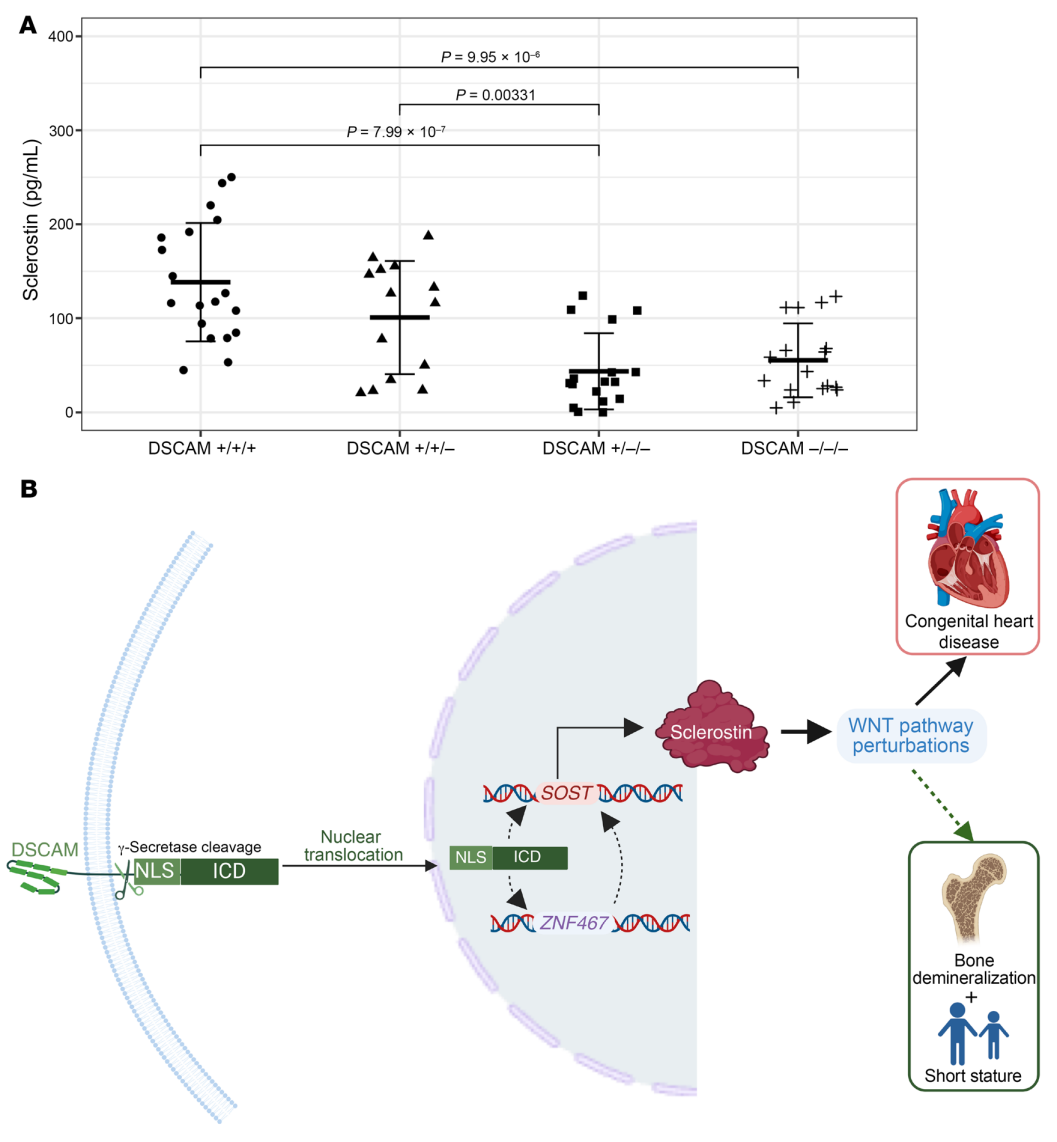
A model for *SOST* activation and Wnt inhibition in T21. (**A**) *DSCAM* is necessary for increased sclerostin secretion in iPSC-derived endothelial cells as measured by an ELISA assay, grouped by *DSCAM* genotypes (+/+/+, *n* = 19; +/+/–, *n* = 14; +/–/–, *n* = 17; –/–/–, *n* = 17 replicates). Error bars represent mean ± SD in each group. *P* values were obtained by 2-tailed Student’s *t* test, with *P* < 0.0083 considered statistically significant following Bonferroni’s correction. Bold bars represent the mean, while the outer bars represent 1 standard deviation. (**B**) Cleavage of DSCAM, followed by nuclear translocation of the intercellular domain (ICD), which contains a nuclear localization signal (NLS), activates transcriptional responses (32). The expression of *DSCAM*, which resides within the chr21 CHD critical region, is correlated with both *ZNF467* (chr1) and *SOST* (chr17) mRNA levels in cardiac endothelial cells from T21 heart tissues. The downstream effects of these molecular signals increase the binding of sclerostin to low-density lipoprotein receptor–related proteins 5 and 6 (LRP5, LRP6) and inhibit Wnt signaling through the frizzled receptor, resulting in attenuated expression of Wnt target genes. Reduced Wnt activity during development could promote CHD, limit skeletal bone maturation resulting in short stature, and increase risks for bone demineralization. Figure created with BioRender.com.

We directly tested the role of *DSCAM* in *SOST* expression by deleting 1, 2, or 3 *DSCAM* alleles in T21 iPSCs using CRISPR/Cas9 homologous recombination (Supplemental Figure 7). From differentiated endothelial cells (iPSC-ECs) we assessed secreted levels of sclerostin using an ELISA and *SOST* and *ZNF461* RNA levels (Supplemental Figure 8). In comparison to the parental T21 iPSC-EC line, disruption of 2 or 3 *DSCAM* alleles resulted in 69% and 60% reduction in sclerostin secretion (*P* = 8.0 × 10^–7^ and *P* = 9.9 × 10^–6^, respectively; Figure 4A). Levels of *SOST* and *ZNF461* transcripts showed high correlations with secreted sclerostin levels (Supplemental Figure 8).

### Downstream effects of increased endothelial cell SOST expression on Wnt signaling.

From snRNA sequencing analyses of CHD, we considered whether *SOST* expression was informative of Wnt activity. Using gene ontology (UniProt, http://uniprot.org) analysis, we identified 65 Wnt target genes (of 410 total) with significantly altered expression (Bonferroni’s threshold *P* < 1.2 × 10^–4^; fold change ≤0.66 or ≥1.5) in T21 versus eCHD endothelial cells (*n* = 65 genes) and atrial cardiomyocytes (68 genes), with overall concordance in both cell lineages (Supplemental Table 19). We observed marked, significant changes in Wnt receptors: *LRP5* (1.8 × 10^–132^) was upregulated in cardiomyocytes, while *LRP4* (*P* < × 10^–250^), with high binding affinity for sclerostin, was downregulated in cardiomyocytes and endothelial cells (22). Expression of *GLI3* (*P* = 1.8 × 10^–54^), dependent on Wnt activity, was reduced in cardiomyocytes (23). Expression of dishevelled gene family members *DVL3* (*P* < 10^–250^) and *DVL1* (*P* = 8.2 × 10^–12^), which transduce canonical Wnt signaling, were increased, as was that of *NPHP3* (*P* = 4.43 × 10^–244^), a primary component of cilia that regulates endothelial-mesenchymal transformation (EMT) and inhibits dishevelled-induced Wnt signaling (24, 25). Increased expression of *AXIN1* (*P* = 1.9 × 10^–9^) and *GSK3α* (*P* = 8.2 × 10^–19^), which regulate cytosolic and nuclear localization of β catenin and the dynamically expressed Wnt inhibitor *DAB2* (*P* = 7.1 × 10^–111^), also implied aberrant Wnt signaling (26, 27).

We also observed increased expression of *IFT80* (*P* = 3.2 × 10^–100^), a primary component of cilia modulating Shh and Wnt signals, and of *HES1* (*P* = 4.0 × 10^–134^), a direct target of Shh and *GLI3* signaling (Supplemental Table 20) (28, 29). Human *IFT80* mutations cause skeletal dysplasia and CHD (29).

## Discussion

By comparing RNA expression in T21 and euploid cardiac tissues and cells, we identified molecular mechanisms contributing to Down syndrome phenotypes. T21 samples showed significantly increased expression for 31 of 100 chr21 protein-coding genes (Supplemental Tables 5 and 7) and 13 of approximately 13,000 protein-coding genes residing on other autosomes (Figure 1 and Table 1). Among these, *SOST* expression was 10-fold higher in T21 compared with euploid tissues; no other RNAs showed differences greater than 2.5-fold. Single-nucleus RNA sequencing analyses and single-RNA molecule in situ hybridization (Figures 2 and 3) demonstrated increased *SOST* expression predominantly in endothelial cells, a lineage that is pervasive across all organs. Both the proportion of endothelial cells expressing *SOST* and the number of *SOST* transcripts per endothelial cell accounted for the increased levels. As *SOST* encodes sclerostin — a secreted inhibitor of Wnt with activities that are essential for the development of the heart and other organs as well as lifelong tissue homeostasis — these data imply that dysregulation of Wnt signaling contributes to multiple T21 phenotypes.

We uncovered a genetic pathway by which T21 increased *SOST* expression, involving a previously reported transcriptional activator, *ZNF467* (16), and *DSCAM*, a chr21 gene encoded in the CHD critical region. Although many chr21 genes were expressed in cardiac endothelial cells, only expression of *DSCAM* correlated with both *ZNF467* and *SOST* (Supplemental Table 18). We showed that *DSCAM* was necessary for *SOST* upregulation in T21 iPSC-ECs (Figure 4A). While *DSCAM* was initially identified as a transmembrane cell adhesion molecule involved in vertebrate and invertebrate neuronal synapse formation (30, 31), two recent studies have indicated broader functions. Intramembrane cleavage of DSCAM protein by γ-secretase releases an intracellular domain with a nuclear localization signal that influences gene expression (32). Additionally, copy number duplications encompassing *DSCAM* were identified in patients with unexplained bicuspid aortic valve and other CHDs (33). Consequently, we propose a regulatory network (Figure 4B) by which increased gene dosage of *DSCAM* increases *ZNF467* expression, which then increases *SOST* expression. However, we do not exclude the possibility that *DSCAM* also directly activates expression of *SOST* and influences dysregulated expression of other Wnt pathway genes (Supplemental Table 19). Notably, as increased *SOST* expression was independent of the age of T21 patients, we expect that activation of this genetic pathway and inhibition of Wnt signaling are lifelong.

Chronic dysregulation of Wnt signaling in cardiomyocytes and endothelial cells could contribute to multiple T21 phenotypes. Developmental Wnt activities are critical for atrioventricular canal progenitors from which the cardiac septa and atrioventricular valves emerge (34–36). Global ablation of *Wnt2* in mice results in high rates of embryonic lethality due to complete atrioventricular canal (37), while *Wnt* silencing at later developmental stages results in valvular defects (38), including AVSD (39). These structures are malformed in 40% of newborns with T21. SHH also participates in atrioventricular septation and may contribute to T21 heart malformations (40, 41). Moreover, genetic ablation of *SOST* both increases Wnt activities and dysregulates SHH signals (42). While we recognize the considerable crosstalk between these pathways, CHD tissues showed only modestly dysregulated expression of SHH target genes (Supplemental Table 20).

Experimental models demonstrate fundamental roles for endothelial cell–derived Wnt signaling in lung and pulmonary vascular development (43) and in the pathophysiology of pulmonary hypertension (44, 45). Thus, sclerostin-mediated attenuation of Wnt activities may contribute to pulmonary hypertension in 45% of T21 patients with CHD and 28% without CHD (46).

Sclerostin secreted by bone endothelial cells and osteoclasts regulates bone remodeling (47): canonical Wnt signaling activates lipoprotein receptor–related proteins (LRP5, LRP6) to promote bone accrual and mineralization, while inactivation of Wnt signaling increases osteoclast differentiation and bone resorption (47). In euploid individuals, low circulating sclerostin levels correlate with pathologically high bone mass (48) and high levels with low bone mass (49). Sclerostin is also a key regulator of craniofacial bone morphology, and human mutations inactivating *SOST* cause sclerosteosis (van Buchem disease), with increased bone mass, mandibular enlargement, and reduced cranial foramen diameter (50). Increased *SOST* expression might therefore cause reciprocal effects: diminutive craniofacial and long bones with low bone density, corresponding to T21 phenotypes of short stature and early-onset osteoporosis-osteopenia (50% of individuals). Importantly, recent preclinical anti-sclerostin antibody treatment in a T21 mouse model prevented bone phenotypes (51).

While our data predict elevated sclerostin levels in all T21 patients, we also suggest that *SOST* eQTLs affect consequences on T21 phenotypes. We expect that all *SOST* eQTLs, including rs6503474, which accounts for approximately 42% of the variance in endothelial expression of *SOST*, will attenuate or augment the increased-dosage effects of T21. rs6503474 is genetically linked to rs1237278 (*r^2^* = 0.893), which resides within a *cis*-regulatory element characterized by a CTCF-binding site overlapping a distal enhancer based on ENCODE annotations (52). By collectively influencing *SOST* expression, these would influence Wnt activities: higher sclerostin levels with greater attenuation of Wnt activities could increase penetrance of a phenotype, while lower sclerostin levels may provide sufficient Wnt activity to suppress T21 phenotypes. Future studies that modulate *DSCAM*, *ZNF467*, and *SOST* in T21 experimental models (53, 54) and correlate circulating sclerostin levels with clinical findings found in individuals with T21 may be informative.

In conclusion, human tissue transcriptional analyses provided new mechanistic insights into how T21 evokes CHD and extracardiac phenotypes. Increasing *SOST* expression would diminish Wnt activities that are critical for the normal development and lifelong homeostasis of multiple tissue with prototypic T21 phenotypes. Validation of these findings may provide therapeutic opportunities for anti-sclerostin antibodies and other strategies that modulate sclerostin activities to benefit T21 phenotypes.

## Methods

### Cohort.

Enrolled PCGC subjects with available discarded CHD tissues with good RNA quality (RIN score ≥5) were studied. Detailed demographic and clinical information on subjects is available through the PCGC data hub (12). Cardiac diagnoses were obtained from review of echocardiogram, catheterization, and operative reports. T21 was ascertained by karyotype. Adult control hearts were obtained and studied as described previously (17).

### Sex as a biological variable.

Tissues from 155 male and 120 female participants were included in the bulk RNA sequencing analysis. Tissues from 12 male and 11 female participants were included in the single-cell nuclear RNA sequencing analysis. Analyses included sex as a biological variable.

### Bulk RNA sequencing.

All CHD tissues were processed for bulk RNA sequencing and analyzed as previously described (11, 17). RNA analyses were studied by site and then genotype. Seven distinct cardiac sites were sampled in at least 6 individual with eCHD and 1 patient with T21. RNA was extracted using TRIzol (Life Technologies). PolyA RNA was extracted and used to generate RNA sequencing libraries as described previously (11). Libraries were sequenced (illumina NextSeq 500, 75-base paired-end reads) to achieve ≥10 million reads (median 30.0, range 8.3–129.5 million reads). Reads were aligned to hg38 reference genome using the STAR aligner (55). Mitochondrial and duplicate reads were discarded using Samtools (56) and Picard MarkDuplicates (Broad Institute), respectively. Gene expression was determined by calculating RPKM.

### Single-nucleus RNA sequencing.

Single-nucleus RNA sequencing was performed from frozen CHD tissues using Cell Ranger 3.1.0 chemistry (10x Genomics) as described previously (57). Euploid adult hearts (17) and fetal developmental atlas (18) were previously reported. Single-nucleus RNA sequencing data were analyzed using the Seurat package in R version 4.0.1 (The R Foundation) as described previously (57). RNA data from nuclei meeting quality metrics were processed as previously reported (17), and data were scaled, batch corrected (58), and clustered before uniform manifold approximation and projection (UMAP) reduction. Cell types were assigned using described designations of coclustered nuclei (17). Single-molecule RNA in situ hybridization was performed as described previously for selected genes (17). Reads per nucleus were compared between T21 and eCHD endothelial cells using 2-sided Student’s *t* test with Bonferroni’s threshold. Coexpression of selected endothelial cell genes were assessed using Pearson’s correlation (*r*).

### Expression quantitative trait locus analyses.

The Genotype-Tissue Expression (GTEx) data used for analyses herein were obtained from the GTEx Portal on May 22, 2022. Previously described eQTLs for *SOST*, identified in GTEx tissues, were used to assess their effects on CHD tissues from patients with paired whole-genome sequences (*n* = 37). We use a linear regression model in the R package Stats, with age and sex as covariates (14). The most substantial eQTL association in T21 tissue was the single nucleotide polymorphism rs6503474; this also modulated *SOST* expression in RA tissues obtained from eCHD patients.

### RNAscope.

Fresh-frozen RA tissues were fixed overnight in 4% paraformaldehyde solution and then placed in 30% sucrose in PBS until they were submerged. Tissues were embedded in O.C.T. compound, sectioned to 5 μm thickness by use of a cryotome, and mounted on Superfrost Plus slides (Thermo Fisher Scientific). Sections were stained by applying the RNAscope version 2.0 assay (Advanced Cell Diagnostics) according to the manufacturer’s protocol and run with positive and negative controls (59). Opal 570 and Opal 650 dyes (Akoya Bioscience) were conjugated to the RNAscope probes targeting *SOST* and *VWF*. Cell membranes were stained with WGA conjugated to Alexa Fluor 488, and nuclei were counterstained with DAPI. Images were acquired on a Leica Stellaris 8 Falcon Dive attached to a fully motorized, upright DM6 microscope base using the single point-scanning confocal imaging modality with a HC PL APO 63×/1.4 DIC oil immersion objective, 405 diode, and white light laser (440–790 nm tunable; 80 Hz pulsed) illumination, Leica Stellaris 8 Power HyD detectors, and a pinhole size of 1 Airy unit (AU). All images were taken sequentially by frame scanning bidirectionally at 400 Hz with a pixel dwell time of 1.04 μs, an image size of 175.74 × 175.74 μm, and a frame size of 1,024 × 1,024. Excitation and emission settings were chosen as the following: DAPI (excitation 405 nm, emission 410–502 nm, HyD S1 detector), Alexa Fluor 488 (excitation 499 nm, emission 504–555 nm, HyDS2 detector), Opal 570 (excitation 552, emission 558–631 nm, HyD S2 detector), and Opal 650 (excitation 628, emission 640–698 nm, HyD R 4 detector), with laser intensities varying between 1% and 5%. Three to 4 images per section were acquired in random locations, and images were edited using Fiji (60).

### Derivation of DSCAM-deficient cell lines.

iPSCs from a patient with T21 were derived from PBMCs as previously described (61). DSCAM CRISPR guide RNA (crRNA, CGGTGGAGGCGTACATCACT) was synthesized as custom Alt-R CRISPR RNA, annealed with transactivating RNA (trcRNA), and incubated with HiFi Cas9 protein to form a functional ribonucleoprotein (RNP) complex following Integrated DNA Technologies’ protocol. RNP complex was transfected to iPSCs, and single-cell clones were obtained as previously described (62). To confirm DSCAM targeting, genomic DNA was isolated from cell pellets using the DNeasy Blood and Tissue Kit (QIAGEN) and PCR amplified with primer forward (ATGAGCCTGTTGACCGTGTG) and reverse (AACCCACCAAAGGATGTCTGAA). Next-generation sequencing libraries were constructed with the Nextera XT DNA Sample Preparation Kit (Illumina) and paired-end sequenced with a read length of 75 base pairs (75PE) on the illumina MiSeq. Reads were aligned to human genome assembly hg38 using the STAR aligner (55). Quantification of allele frequency was performed using CrispRVariants (Bioconductor) (63).

### iPSC endothelial cell differentiation.

iPSCs were maintained in 6-well plates with daily exchange of mTeSR-1 medium (85850, StemCell Technologies). Endothelial cell differentiation (ECD) was started when cells reached 70%–80% confluency. Briefly, on day 0, iPSCs were switched to RPMI medium (11879-020, Invitrogen) supplemented with B27 minus insulin (A1895601, Thermo Fisher Scientific) and 7 μM CHIR99021 a glycogen synthase kinase 3 (GSK-3) activator/Wnt inhibitor (44-231-0, Tocris). Medium was exchanged on day 2 with RPMI/B27 minus insulin medium supplemented with 4 μM CHIR99021 and 10 μM SB431542, a TGF-β, ALK4, and AhLK7 inhibitor (72234, StemCell Technologies). On day 4, medium was exchanged with Endothelial Cell Growth Medium-2 (EGM-2, CC-3162, Lonza) supplemented with 10 μM SB431542, 50 ng/mL VEGF (VE-293-010, R&D Systems), and 25 ng/mL FGF (233-FB-025, R&D Systems). On day 6 and day 8 of ECD, medium was exchanged with EGM-2 medium supplemented with 50 ng/mL VEGF and 25 ng/mL FGF. From day 10 onward, medium was exchanged every other day with EGM-2 medium.

### Endothelial cell purification.

Between day 9 and day 12 of ECD, endothelial cells were purified by FACS. Briefly, cells were detached with Accutase (07920, StemCell Technologies) and washed once with FACS buffer–PBS (10010-049, Invitrogen) supplemented with 0.5% BSA (A9418, Sigma-Aldrich). Next, cells were stained with anti-CD144 (VE-cadherin) monoclonal antibody (14-1449-82, Thermo Fisher Scientific) for 1 hour at 4°C. Cells were then washed 3 times with FACS buffer and stained with a fluorescent secondary antibody (ab150113, Abcam) for 30 minutes at 4°C. Finally, cells were washed 3 times with FACS buffer prior to cell sorting. Following sorting, endothelial cells were replated onto a 6-well plate in EGM-2 medium supplemented with 10 μM Y-27631, a Rho kinase inhibitor (125410, R&D Systems). Medium was exchanged with EGM-2 medium the following day and every other day thereafter.

### Quantification of SOST secretion from iPSCs.

Once enriched endothelial cells reached 100% confluency, conditioned media were collected for quantification of SOST secretion by ELISA (ab221836, Abcam) following the manufacturer’s instructions.

### Statistics.

A tissue-specific *z* score for each T21 sample against all eCHD samples was calculated. From aggregated *z* scores, grouped by genotype (T21 or eCHD), Student’s 2-tailed *t* test *P* values were calculated. *P* value cutoffs were Bonferroni corrected by the number of differentially expressed genes (RPKM ≥2.5) encoded on chr21 (4.8 × 10^–4^) or the total on other chromosomes (4.1 × 10^–6^). An average difference in gene expression of ≥1.5-fold or ≤0.66-fold was considered significant.

Quantification of the total number of endothelial cells (*VWF* positive) and *SOST*-expressing endothelial cells (*VWF* and *SOST* positive) was performed manually using the Cell Counter plugin in Fiji (60). Cells were counted in 4 images per individual of 4 T21 and 3 eCHD subjects (a total of 16 images for T21 and 12 images for eCHD; images from2 sections per subject). Endothelial cells were considered as *SOST* expressing if either an overlay of *VWF* and *SOST* could be observed or the *SOST* signal was located within the cell boundaries delineated by WGA. Cell proportions and signal counts between T21 and eCHD were compared using the 2-tailed *t* test with *P* < 0.05 set as the significance threshold.

### Study approval.

Individuals with Down syndrome, those with CHD, and other CHD probands were recruited into the Congenital Heart Disease Genetic Network Study of the Pediatric Cardiac Genomics Consortium (CHD GENES; ClinicalTrials.gov NCT01196182). The protocol was approved by the Institutional Review Boards of Boston Children’s Hospital, Brigham and Women’s Hospital, Great Ormond Street Hospital for Children, Children’s Hospital of Los Angeles, Children’s Hospital of Philadelphia, Columbia University Medical Center, Icahn School of Medicine at Mount Sinai, University of Rochester School of Medicine and Dentistry, Steven and Alexandra Cohen Children’s Medical Center of New York, and Yale School of Medicine. Each participating individual or their parent/guardian provided written informed consent. Probands with CHD and individuals with Down syndrome were selected based on availability of cardiovascular tissue that had been discarded during corrective surgery. Blood samples were obtained after patients were enrolled and provided consent.

### Data availability.

All underlying data for figures are provided in the Supporting data values file. Additional participant data are available through the PCGC data hub (12); and to investigators with Institutional Review Board–approved dbGaP access (phs000571.v6.p2, RNA-Seq). Bulk RNA sequencing data are available through the NCBI’s Gene Expression Omnibus database (GEO GSE253244).

## Author contributions

DMM, JGS, and CES designed the study. PCGC investigators recruited and enrolled CHD patients and with JDC, RK, and BM acquired CHD tissues. Primary authors are designated based on their development and analyses of key resources used throughout the study. DMD, SRD, JMG, and JAW performed RNA sequencing and bioinformatics processing. DMM, PN, SUM, VS, SM, DQ, QZ, DR, KB, MYJ, TW, CES, and JGS performed computational analyses. PN, HW, KB, DR, VTT, AS, and VS performed confirmational experiments. DMM, PN, SM, DQ, SUM, VTT, VS, JGS, CES drafted the manuscript, and all authors contributed to editing the manuscript.

## Supplementary Material

Supplemental data

Supplemental tables 1-20

Supporting data values

## Figures and Tables

**Table 1:**
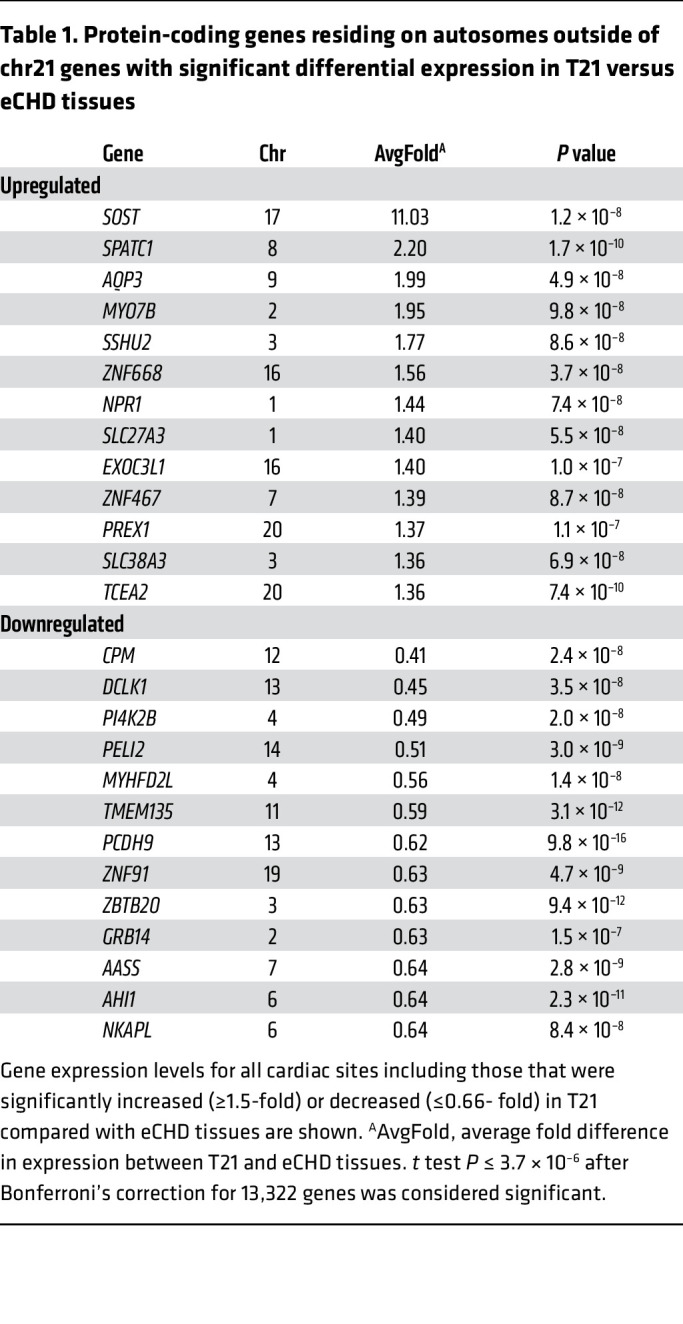
Protein-coding genes residing on autosomes outside of chr21 genes with significant differential expression in T21 versus eCHD tissues

## Version 1 06/03/2024

Electronic publication
