# Supplementary material for: Increased endothelial sclerostin caused by elevated DSCAM mediates multiple trisomy 21 phenotypes
Source: J Clin Invest. 2024 Jun 3;134(11):e167811. doi: 10.1172/JCI167811 (PMC11142749; doi:10.1172/JCI167811)
Supplement: Supplemental data [file jci-134-167811-s125.pdf]

SUPPLEMENTARY APPENDIX

Table of Contents

Supplementary Figures	2-9
Supplementary Tables	See Data Supplement
References	10-14
Supplementary Acknowledgements	15

SUPPLEMENTAL FIGURES

Supplementary Figure 1

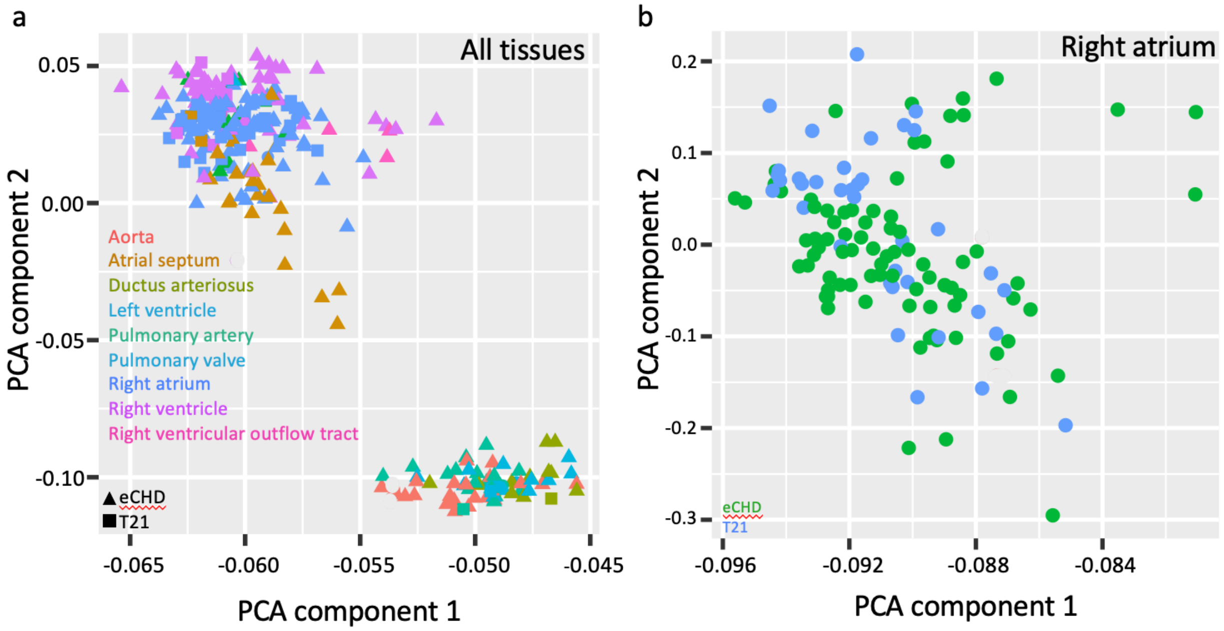

Supplementary Figure 1: Principal Component Analyses (PCA) of RNAseq data from CHD tissues. Panel A shows PCA of RNAseq data from different CHD tissues (color coded) distinguished myocardial samples (right atria, atrial septum, right ventricle, and outflow tract, left ventricle) from extra-cardiac (aorta, ductus arteriosus, pulmonary artery, and valve) tissues but not eCHD (triangle) and T21 (box) specimens, indicating similar global transcriptomes. Panel B shows PCA of right atrial tissues confirm similar global transcriptomes of eCHD and T21 tissues.

Supplementary Figure 2

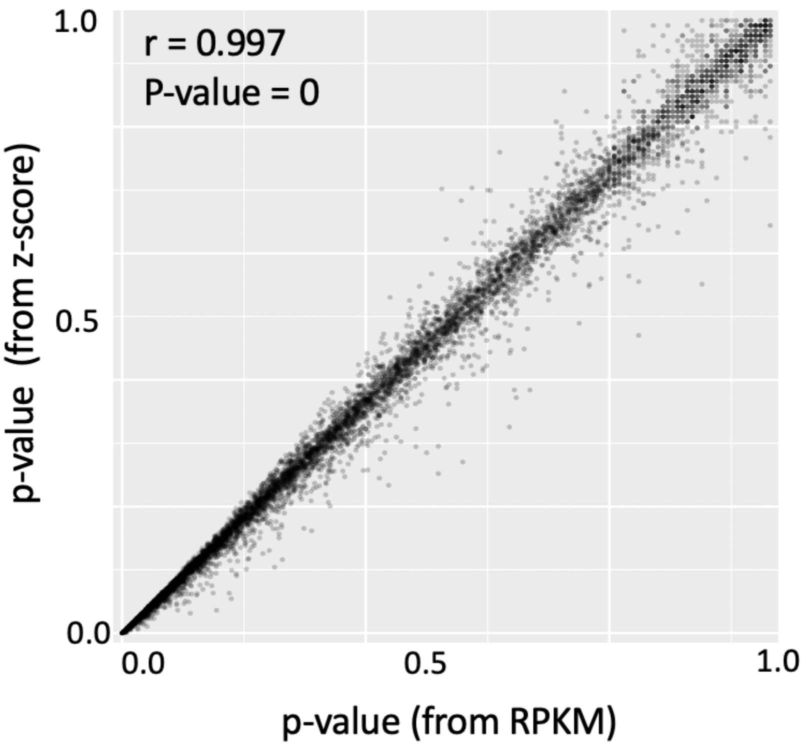

Supplementary Figure 2: Expression level of genes, assessed by RPKM or standard (Z-) scores and stratified by adjusted p-values (adjP), in T21 compared to eCHD right atrial tissues.

Supplementary Figure 3

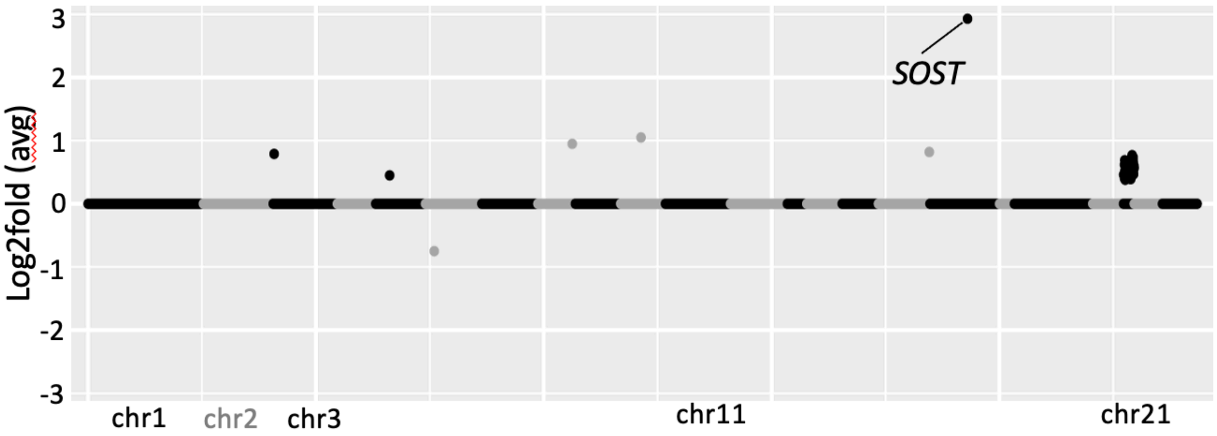

Supplementary Figure 3: Graphic representation showing the chromosome location of genes with significant differential gene expression in T21 versus eCHD RA tissues.
Five genes encoded outside of chr21 showed significant (fold≥1.5 or fold≤0.5; p< 4.1E-6) differential expression: *SOST. OIT3, SPATC1, MC1R, SSUH2, F2R* and *NKAPL*. On chr21, 63 genes showed significantly different expression (Supplementary Table 5).

Supplementary Figure 4

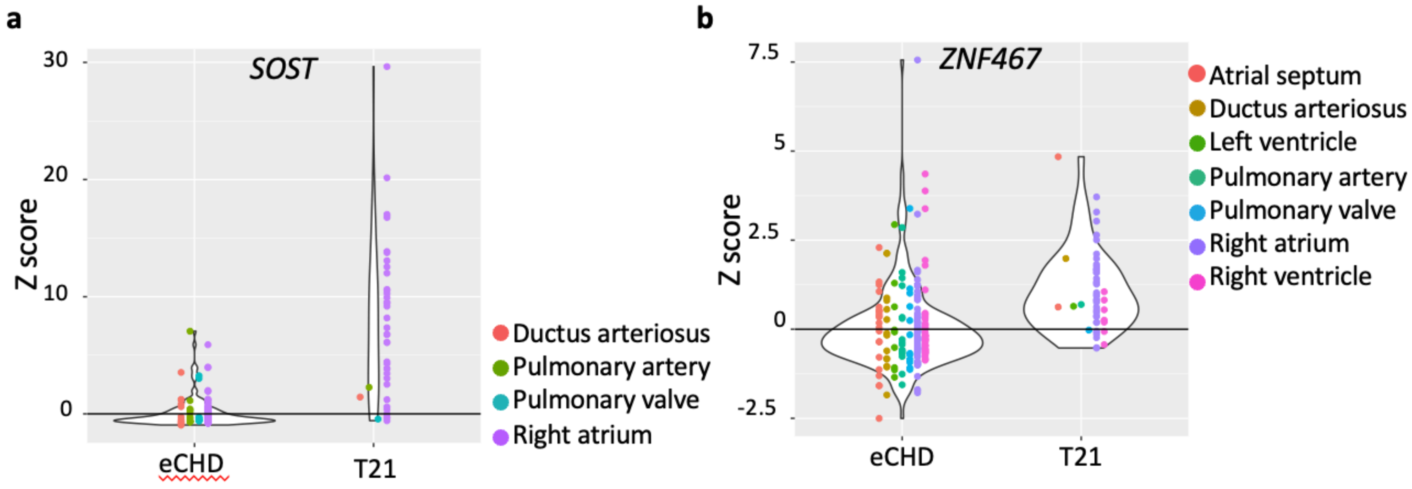

Supplementary Figure 4: Comparison of standard expression of SOST1 (a) and ZNF467 (b) transcripts in eCHD and T21 tissues.
Standard expression (Z-scores) is plotted by tissue origin**.** Abbreviations PA, pulmonary artery; PV, pulmonary valve; RA, right atria, AtrSpt, atrial septum, DuctArt, ductus arteriosus LV, left ventricle, RV, right ventricle**.**

Supplementary Figure 5

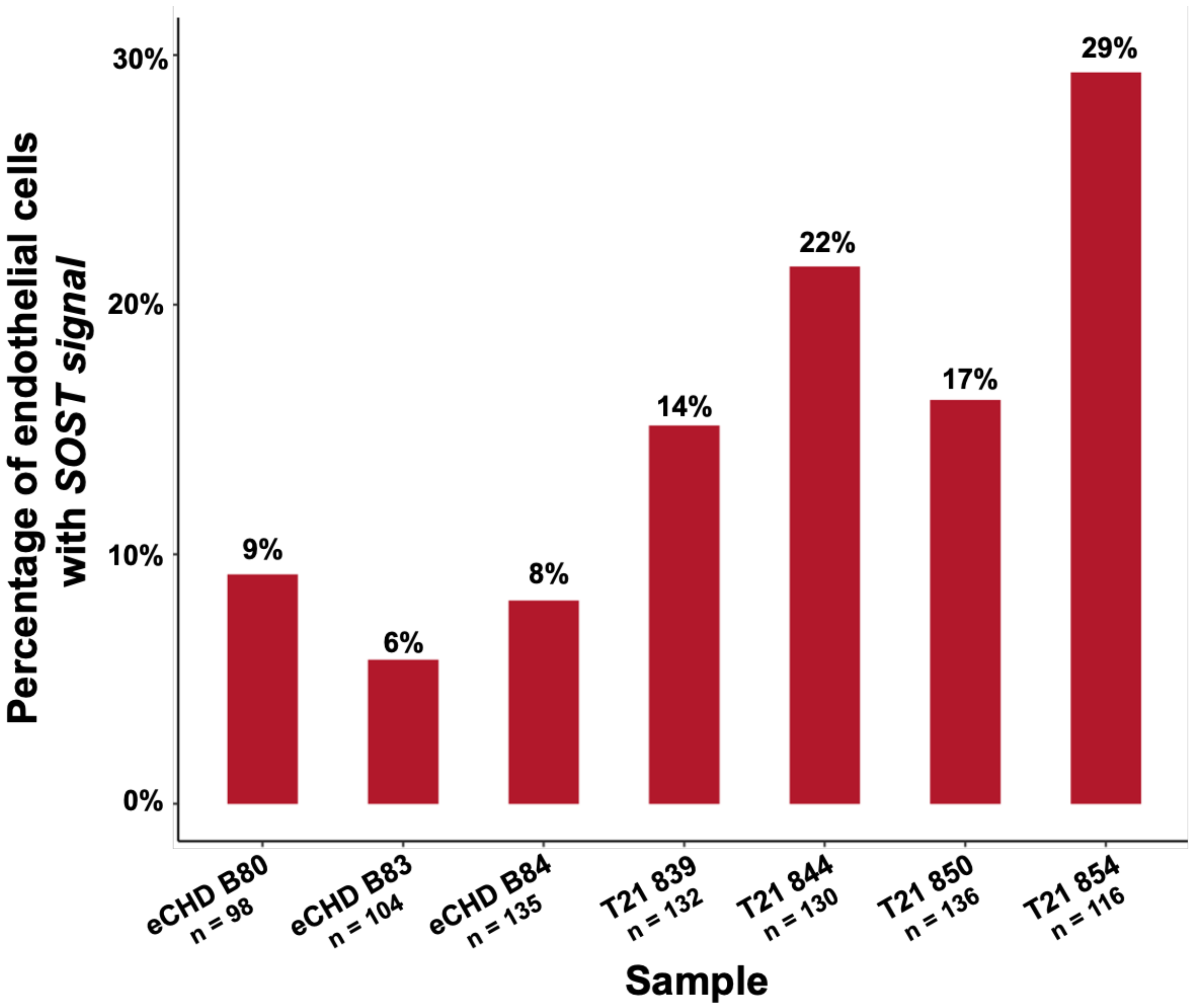

Supplementary Figure 5: Proportion of endothelial cells expressing *SOST* as determined by RNAscope.
Number (n) below each sample bar indicates the total number of endothelial cells counted.

Supplementary Figure 6

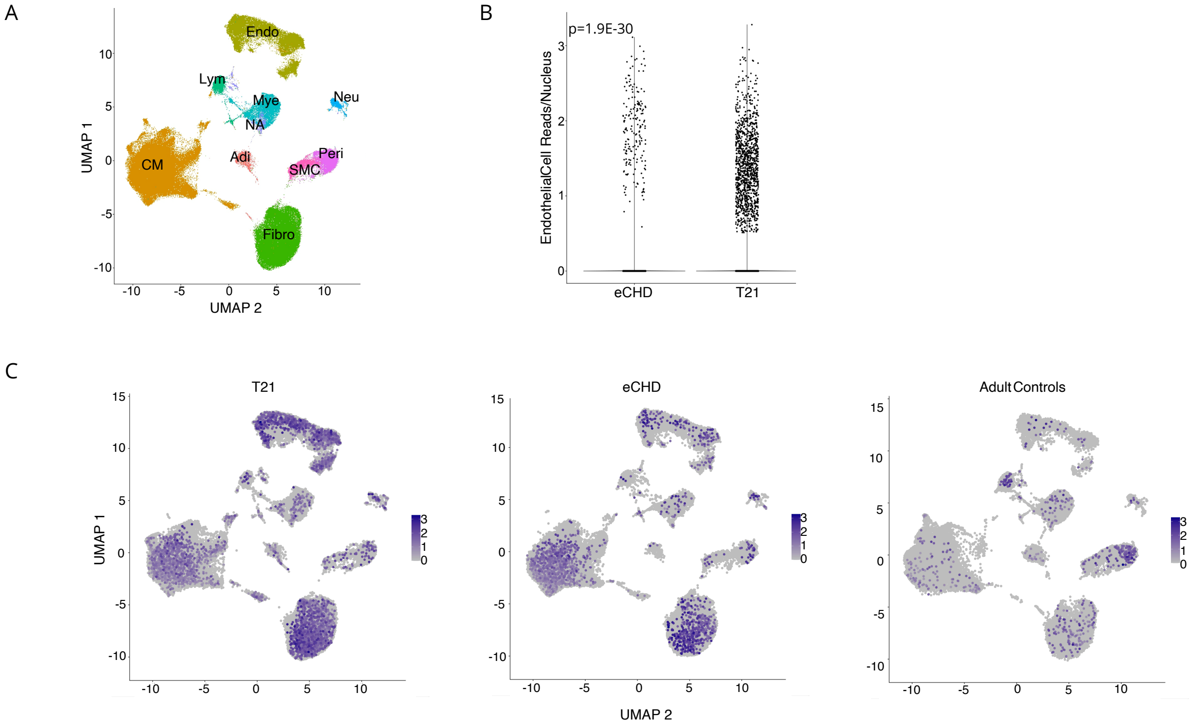

Supplementary Figure 6: snRNAseq *C21orf62* Expression Analysis in Cardiac Tissue
Panel A shows a uniform manifold and projection graph of single-nuclear RNA-sequencing right atria from T21 (n=10), eCHD (n=13), and healthy adults (n=12) identifies eight major cell types (Adi, adipocytes; CM, atrial cardiomyocytes; Endo, endothelial cells; Fibro, fibroblasts; Lym, lymphocytes; Mye, myeloid cells; NA, not assigned; Neu, neural cells; Peri, pericytes). Panel B shows feature plots feature plots that demonstrate the variable expression of *C21orf62* in T21, eCHD, and healthy adult right atria. Note different scales in heat maps. Panel C shows comparison of the endothelial cell transcript levels for *C21orf62* expression in T21 and eCHD samples.

Supplementary Figure 7

DSCAM+/+/+

Allele	Genotype	Sequence
1	Reference	940 CCTCCTCGGTGGAGGCGTACATCACT 965
2	Reference	940 CCTCCTCGGTGGAGGCGTACATCACT 965
3	Reference	940 CCTCCTCGGTGGAGGCGTACATCACT 965

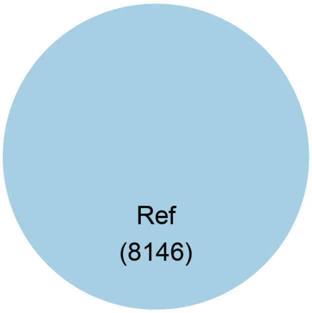

DSCAM+/+/-

Allele	Genotype	Sequence
1	Reference	940 CCTCCTCGGT-GGAGGCGTACATCACT 965
2	Reference	940 CCTCCTCGGT-GGAGGCGTACATCACT 965
3	c.949_950insT	940 CCTCCTCGGT**T**GGAGGCGTACATCACT 965

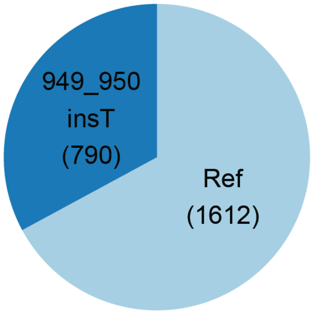

DSCAM+/-/-

Allele	Genotype	Sequence
1	Reference	940 CCTCCTCGGT-GGAGGCGTACATCACT 965
2	c.949_950insT	940 CCTCCTCGGT**T**GGAGGCGTACATCACT 965
3	c.949_950insT	940 CCTCCTCGGT**T**GGAGGCGTACATCACT 965

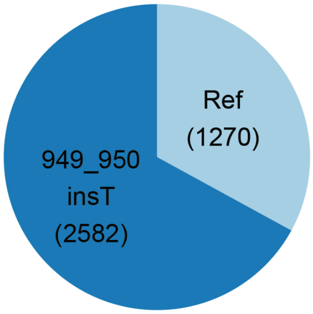

DSCAM-/-/-

Allele	Genotype	Sequence
1	c.949_950insTT	940 CCTCCTCGGT**TT**GGAGGCGTACATCACT 965
2	c.944_962del	940 CCTC----------------------ACT 965
3	c.945_949del	940 CCTCCT------GGAGGCGTACATCACT 965

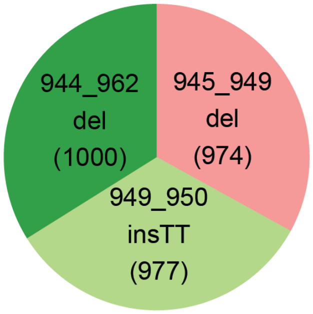

Supplementary Figure 7: DSCAM genotypes of iPSC lines. Variants were annotated based on the *DSCAM* mRNA reference sequence (Accession number NM_001271534.3). Pie charts on the right show the proportion of each allele based MiSeq. Numbers in parentheses denote the read count corresponding to each allele.

Supplementary Figure 8

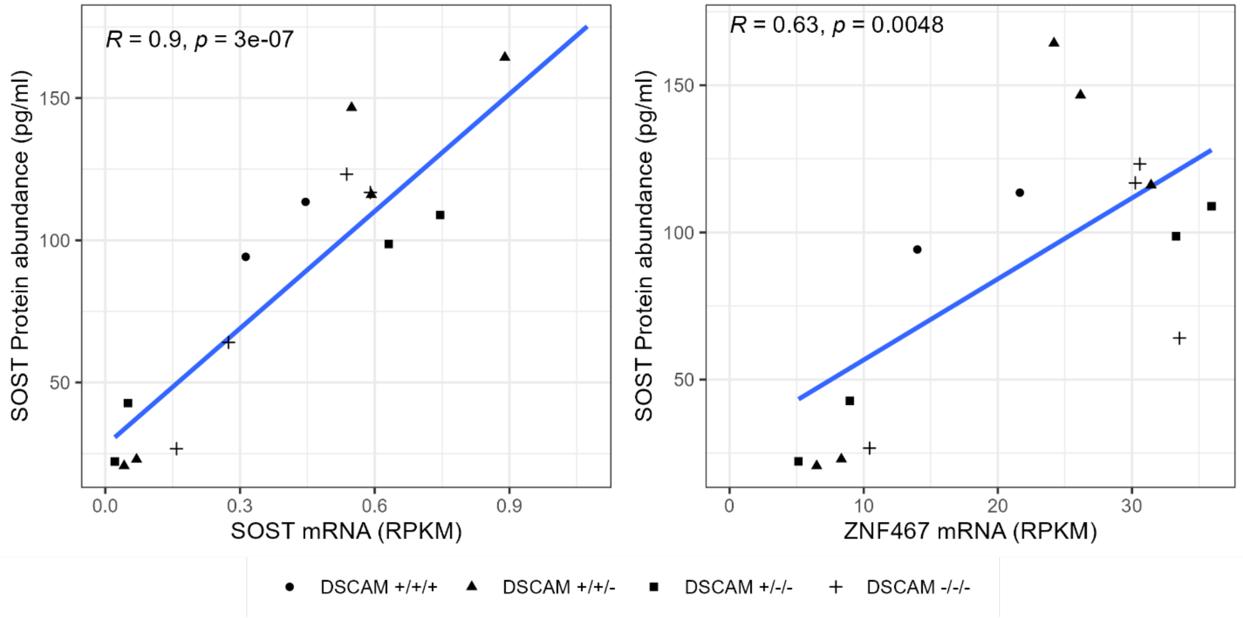

Supplementary Figure 8: High correlation between *SOST* and *ZNF467* mRNA levels and SOST abundance in the media across genotypes in iPSC-ECs. *SOST* and *ZNF467* mRNA levels were determined by bulk RNA-sequencing. SOST protein abundance was determined by an ELISA assay.

References

1. Antonarakis SE, et al. Down syndrome. *Nat Rev Dis Primers*. 2020;6(1):9. doi:10.1038/s41572-019-0143-7

2. Bergström S, et al. Trends in Congenital Heart Defects in Infants With Down Syndrome. *Pediatrics*. 2016;138(1):e20160123. doi:10.1542/peds.2016-0123

3. Bull MJ. Down Syndrome. *N Engl J Med*. 2020;382(24):2344-2352. doi:10.1056/NEJMra1706537

4. Pestel J, et al. Real-time 3D visualization of cellular rearrangements during cardiac valve formation. *Development*. 2016;143(12):2217-2227. doi:10.1242/dev.133272

5. Tan M, et al. Types and distribution of congenital heart defects associated with trisomy 21 in Singapore. *J Paediatr Child Health*. 2013;49(3):223-227. doi:10.1111/jpc.12129

6. Homsy J, et al. De novo mutations in congenital heart disease with neurodevelopmental and other congenital anomalies. *Science*. 2015;350(6265):1262-1266. doi:10.1126/science.aac9396

7. Lana-Elola E, et al. Genetic dissection of Down syndrome-associated congenital heart defects using a new mouse mapping panel. *Elife*. 2016;5. doi:10.7554/eLife.11614

8. Pelleri MC, et al. Genotype-phenotype correlation for congenital heart disease in Down syndrome through analysis of partial trisomy 21 cases. *Genomics*. 2017;109(5-6):391-400. doi:10.1016/j.ygeno.2017.06.004

9. Pelleri MC, et al. Integrated Quantitative Transcriptome Maps of Human Trisomy 21 Tissues and Cells. *Front Genet*. 2018;9:125. doi:10.3389/fgene.2018.00125

10. Li C, et al. Genome-wide expression analysis in Down syndrome: insight into immunodeficiency. *PLoS One*. 2012;7(11):e49130. doi:10.1371/journal.pone.0049130

11. McKean DM, et al. Loss of RNA expression and allele-specific expression associated with congenital heart disease. *Nat Commun*. 2016;7:12824. doi:10.1038/ncomms12824

12. Pediatric Cardiac Genomics C, et al. The Congenital Heart Disease Genetic Network Study: rationale, design, and early results. *Circ Res*. 2013;112(4):698-706. doi:10.1161/CIRCRESAHA.111.300297

13. Chen S, et al. *A Genome-Wide Mutational Constraint Map Quantified from Variation in 76,156 Human Genomes*. Genetics; 2022. doi:10.1101/2022.03.20.485034

14. G. TEx Consortium. The Genotype-Tissue Expression (GTEx) project. *Nat Genet*. 2013;45(6):580-585. doi:10.1038/ng.2653

15. The GTEx Consortium. The GTEx Consortium atlas of genetic regulatory effects across human tissues. *Science*. 2020;369(6509):1318-1330. doi:10.1126/science.aaz1776

16. You L, et al. Zinc finger protein 467 regulates Wnt signaling by modulating the expression of sclerostin in adipose derived stem cells. *Biochem Biophys Res Commun*. 2015;456(2):598-604. doi:10.1016/j.bbrc.2014.11.120

17. Litviňuková M, et al. Cells of the adult human heart. *Nature*. 2020;588(7838):466-472. doi:10.1038/s41586-020-2797-4

18. Cao J, et al. A human cell atlas of fetal gene expression. *Science*. 2020;370(6518):eaba7721. doi:10.1126/science.aba7721

19. de Soysa TY, et al. Single-cell analysis of cardiogenesis reveals basis for organ-level developmental defects. *Nature*. 2019;572(7767):120-124. doi:10.1038/s41586-019-1414-x

20. Smedley D, et al. The BioMart community portal: an innovative alternative to large, centralized data repositories. *Nucleic Acids Res*. 2015;43(W1):W589-W598. doi:10.1093/nar/gkv350

21. Grossman TR, et al. Over-expression of DSCAM and COL6A2 cooperatively generates congenital heart defects. *PLoS Genet*. 2011;7(11):e1002344. doi:10.1371/journal.pgen.1002344

22. Katchkovsky S, et al. Competitive blocking of LRP4–sclerostin binding interface strongly promotes bone anabolic functions. *Cell Mol Life Sci*. 2022;79(2):113. doi:10.1007/s00018-022-04127-2

23. Alvarez-Medina R, et al. Wnt canonical pathway restricts graded Shh/Gli patterning activity through the regulation of Gli3 expression. *Development*. 2008;135(2):237-247. doi:10.1242/dev.012054

24. Egorova AD, et al. Lack of Primary Cilia Primes Shear-Induced Endothelial-to-Mesenchymal Transition. *Circ Res*. 2011;108(9):1093-1101. doi:10.1161/CIRCRESAHA.110.231860

25. Bergmann C, et al. Loss of nephrocystin-3 function can cause embryonic lethality, Meckel-Gruber-like syndrome, situs inversus, and renal-hepatic-pancreatic dysplasia. *Am J Hum Genet*. 2008;82(4):959-970. doi:10.1016/j.ajhg.2008.02.017

26. Wang Z, Li Z, Ji H. Direct targeting of *β* -catenin in the Wnt signaling pathway: Current progress and perspectives. *Medicinal Research Reviews*. 2021;41(4):2109-2129. doi:10.1002/med.21787

27. Hofsteen P, et al. Quantitative proteomics identify DAB2 as a cardiac developmental regulator that inhibits WNT/β-catenin signaling. *Proc Natl Acad Sci USA*. 2016;113(4):1002-1007. doi:10.1073/pnas.1523930113

28. Sharma D, et al. HES1 is a novel downstream modifier of the SHH-GLI3 Axis in the development of preaxial polydactyly. Long F, ed. *PLoS Genet*. 2021;17(12):e1009982. doi:10.1371/journal.pgen.1009982

29. Cavalcanti DP, et al. Mutation in IFT80 in a fetus with the phenotype of Verma-Naumoff provides molecular evidence for Jeune-Verma-Naumoff dysplasia spectrum. *Journal of Medical Genetics*. 2011;48(2):88-92. doi:10.1136/jmg.2009.069468

30. Lowe SA, Hodge JJL, Usowicz MM. A third copy of the Down syndrome cell adhesion molecule (Dscam) causes synaptic and locomotor dysfunction in Drosophila. *Neurobiol Dis*. 2018;110:93-101. doi:10.1016/j.nbd.2017.11.013

31. Liu H, et al. DSCAM gene triplication causes excessive GABAergic synapses in the neocortex in Down syndrome mouse models. *PLoS Biol*. 2023;21(4):e3002078. doi:10.1371/journal.pbio.3002078

32. Sachse SM, et al. Nuclear import of the DSCAM-cytoplasmic domain drives signaling capable of inhibiting synapse formation. *EMBO J*. 2019;38(6):e99669. doi:10.15252/embj.201899669

33. Carlisle SG, et al. *Rare Genomic Copy Number Variants Implicate New Candidate Genes for Bicuspid Aortic Valve*. Genetic and Genomic Medicine; 2023. doi:10.1101/2023.10.23.23297397

34. Briggs LE, et al. Wnt/β-catenin and sonic hedgehog pathways interact in the regulation of the development of the dorsal mesenchymal protrusion. *Developmental Dynamics*. 2016;245(2):103-113. doi:10.1002/dvdy.24339

35. Hurlstone AFL, et al. The Wnt/beta-catenin pathway regulates cardiac valve formation. *Nature*. 2003;425(6958):633-637. doi:10.1038/nature02028

36. Combs MD, Yutzey KE. Heart valve development: regulatory networks in development and disease. *Circ Res*. 2009;105(5):408-421. doi:10.1161/CIRCRESAHA.109.201566

37. Tian Y, et al. Characterization and In Vivo Pharmacological Rescue of a Wnt2-Gata6 Pathway Required for Cardiac Inflow Tract Development. *Developmental Cell*. 2010;18(2):275-287. doi:10.1016/j.devcel.2010.01.008

38. Gillers BS, et al. Canonical Wnt Signaling Regulates Atrioventricular Junction Programming and Electrophysiological Properties. *Circ Res*. 2015;116(3):398-406. doi:10.1161/CIRCRESAHA.116.304731

39. Verhoeven MC, et al. Wnt signaling regulates atrioventricular canal formation upstream of *BMP* and *Tbx2*. *Birth Defects Research*. 2011;91(6):435-440. doi:10.1002/bdra.20804

40. Moyer AJ, et al. Overexpression screen of chromosome 21 genes reveals modulators of Sonic hedgehog signaling relevant to Down syndrome. *Dis Model Mech*. 2023;16(4):dmm049712. doi:10.1242/dmm.049712

41. Gabriel GC, Young CB, Lo CW. Role of cilia in the pathogenesis of congenital heart disease. *Seminars in Cell & Developmental Biology*. 2021;110:2-10. doi:10.1016/j.semcdb.2020.04.017

42. Collette NM, et al. Sost and its paralog Sostdc1 coordinate digit number in a Gli3-dependent manner. *Dev Biol*. 2013;383(1):90-105. doi:10.1016/j.ydbio.2013.08.015

43. Vladar EK, Königshoff M. Noncanonical Wnt planar cell polarity signaling in lung development and disease. *Biochemical Society Transactions*. 2020;48(1):231-243. doi:10.1042/BST20190597

44. Yuan K, et al. Loss of Endothelium-Derived Wnt5a Is Associated With Reduced Pericyte Recruitment and Small Vessel Loss in Pulmonary Arterial Hypertension. *Circulation*. 2019;139(14):1710-1724. doi:10.1161/CIRCULATIONAHA.118.037642

45. de Jesus Perez V, et al. Targeting the Wnt signaling pathways in pulmonary arterial hypertension. *Drug Discovery Today*. 2014;19(8):1270-1276. doi:10.1016/j.drudis.2014.06.014

46. Bush D, et al. Clinical Characteristics and Risk Factors for Developing Pulmonary Hypertension in Children with Down Syndrome. *J Pediatr*. 2018;202:212-219.e2. doi:10.1016/j.jpeds.2018.06.031

47. Delgado-Calle J, Sato AY, Bellido T. Role and mechanism of action of sclerostin in bone. *Bone*. 2017;96:29-37. doi:10.1016/j.bone.2016.10.007

48. Balemans W, et al. Increased bone density in sclerosteosis is due to the deficiency of a novel secreted protein (SOST). *Hum Mol Genet*. 2001;10(5):537-543.

49. Yorgan TA, et al. The Anti-Osteoanabolic Function of Sclerostin Is Blunted in Mice Carrying a High Bone Mass Mutation of Lrp5. *J Bone Miner Res*. 2015;30(7):1175-1183. doi:10.1002/jbmr.2461

50. Sebastian A, Loots GG. Genetics of Sost/SOST in sclerosteosis and van Buchem disease animal models. *Metabolism*. 2018;80:38-47. doi:10.1016/j.metabol.2017.10.005

51. Williams DK, et al. Sclerostin Antibody Treatment Stimulates Bone Formation to Normalize Bone Mass in Male Down Syndrome Mice. *JBMR Plus*. 2018;2(1):47-54. doi:10.1002/jbm4.10025

52. The ENCODE Project Consortium, et al. Expanded encyclopaedias of DNA elements in the human and mouse genomes. *Nature*. 2020;583(7818):699-710. doi:10.1038/s41586-020-2493-4

53. Komori T. Mouse Models for the Evaluation of Osteocyte Functions. *J Bone Metab*. 2014;21(1):55. doi:10.11005/jbm.2014.21.1.55

54. Zheng S, et al. Sclerostin aggravates cardiac remodeling after myocardial infarction by inhibition of Wnt/β-catenin signaling pathway. *J Thorac Dis*. 2022;14(5):1563-1577. doi:10.21037/jtd-22-473

55. Dobin A, et al. STAR: ultrafast universal RNA-seq aligner. *Bioinformatics*. 2013;29(1):15-21. doi:10.1093/bioinformatics/bts635

56. Li H, et al. The Sequence Alignment/Map format and SAMtools. *Bioinformatics*. 2009;25(16):2078-2079. doi:10.1093/bioinformatics/btp352

57. Nadelmann ER, et al. Isolation of Nuclei from Mammalian Cells and Tissues for Single-Nucleus Molecular Profiling. *Current Protocols*. 2021;1(5). doi:10.1002/cpz1.132

58. Korsunsky I, et al. Fast, sensitive and accurate integration of single-cell data with Harmony. *Nat Methods*. 2019;16(12):1289-1296. doi:10.1038/s41592-019-0619-0

59. Wang F, et al. RNAscope. *The Journal of Molecular Diagnostics*. 2012;14(1):22-29. doi:10.1016/j.jmoldx.2011.08.002

60. Schindelin J, et al. Fiji: an open-source platform for biological-image analysis. *Nat Methods*. 2012;9(7):676-682. doi:10.1038/nmeth.2019

61. Oliveira TGM, et al. Different Transcriptomic Response to T. cruzi Infection in hiPSC-Derived Cardiomyocytes From Chagas Disease Patients With and Without Chronic Cardiomyopathy. *Front Cell Infect Microbiol*. 2022;12:904747. doi:10.3389/fcimb.2022.904747

62. Agarwal R, et al. Pathogenesis of Cardiomyopathy Caused by Variants in *ALPK3* , an Essential Pseudokinase in the Cardiomyocyte Nucleus and Sarcomere. *Circulation*. 2022;146(22):1674-1693. doi:10.1161/CIRCULATIONAHA.122.059688

63. Lindsay H, et al. CrispRVariants charts the mutation spectrum of genome engineering experiments. *Nat Biotechnol*. 2016;34(7):701-702. doi:10.1038/nbt.3628

Supplementary Acknowledgements

Pediatric Cardiac Genomics Consortium investigators and affiliations:

Casey A. Gifford, Ph.D.[1,2], T. Yvanka de Soysa, Ph.D. [1,2], Daniel Bernstein[3], M.D., Martina Brueckner, M.D.[4], Benoit G. Bruneau, Ph.D. [1,2,5], Bruce D. Gelb, M.D.[6], Elizabeth Goldmuntz, M.D.[7], Richard P. Lifton, M.D., Ph.D.[4,8], Jane W. Newburger, M.D., M.P.H.[9,10], Amy E. Roberts, M.D. [9,10], Deepak Srivastava, M.D.[1,2,5], H. Joseph Yost, Ph.D.[11], Martin Tristani-Firouz, M.D.[12], Alexander G. Robling, Ph.D.[13], Richard N. Mitchell, M.D., Ph.D.[14], Wendy K. Chung, M.D.[15]

[1]Gladstone Institutes, San Francisco, CA, USA

[2]Roddenberry Center for Stem Cell Biology and Medicine, Gladstone Institutes, San Francisco, CA, USA

[3]Department of Pediatrics, Stanford University School of Medicine, CA USA.

[4]Departments of Genetics and Pediatrics, Yale University School of Medicine, New Haven, CT, USA.

[5]Department of Pediatrics and Cardiovascular Research Institute, UCSF, San Francisco, CA, USA.

[6]Mindich Child Health and Development Institute, Icahn School of Medicine at Mount Sinai, New York, NY, USA; Department of Genetics and Genomic Sciences, Icahn School of Medicine at Mount Sinai, New York, NY, USA; Department of Pediatrics, Icahn School of Medicine at Mount Sinai, New York, NY, 10029, USA.

[7]Department of Pediatrics, The Perelman School of Medicine, University of Pennsylvania, Philadelphia, PA, USA

[8]Howard Hughes Medical Institute, Yale University, New Haven, CT, 06510, USA; Yale Center for Mendelian Genomics, New Haven, CT, USA; Yale Center for Genome Analysis, Yale University, New Haven, CT, USA; Department of Internal Medicine, Yale University School of Medicine, New Haven, CT, USA.

[9]Department of Pediatrics, Harvard Medical School, Boston, MA, USA.

[10]Department of Cardiology, Boston Children's Hospital, Boston, MA, USA.

[11]Molecular Medicine Program, Eccles Institute of Human Genetics, University of Utah and School of Medicine, Salt Lake City, UT, USA.

[12]Division of Pediatric Cardiology, University of Utah and School of Medicine, Salt Lake City, UT, USA.

[13]Department of Anatomy and Cell Biology, Indiana University School of Medicine, Indianapolis, IN, USA; Richard L. Roudebush VA Medical Center, Indianapolis, IN, USA; Department of Biomedical Engineering, Indiana University-Purdue University at Indianapolis, Indianapolis, IN, USA.

[14]Department of Pathology, Brigham and Women's Hospital, Boston, MA, USA.

[15]Departments of Pediatrics and Medicine, Columbia University Medical Center, New York, NY, USA.
